# Supplementary figures and images for: Characterization and clustering of kinase isoform expression in metastatic melanoma
Source: PLoS Comput Biol. 2022 May 13;18(5):e1010065. doi: 10.1371/journal.pcbi.1010065 (PMC9132324; doi:10.1371/journal.pcbi.1010065)

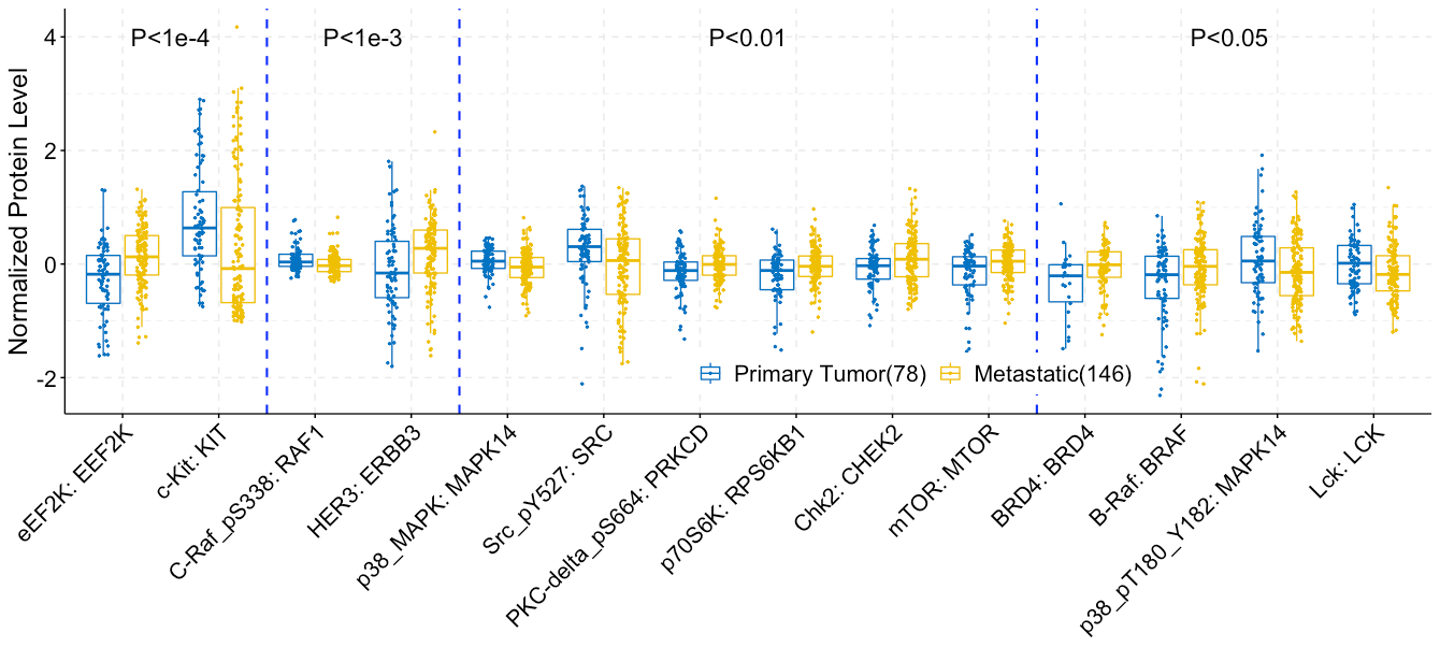

Supplement: S1 Fig — Wilcoxon’s rank-sum test was performed on 208 protein probes between 78 primary and 146 metastatic high purity tumor samples. Shown are 14 kinases that were significant at the level of BH adjusted p-value<0.05. X-axis labels indicate the RPPA probe and the corresponding gene encoding that protein. (TIF) [file pcbi.1010065.s001.tif]

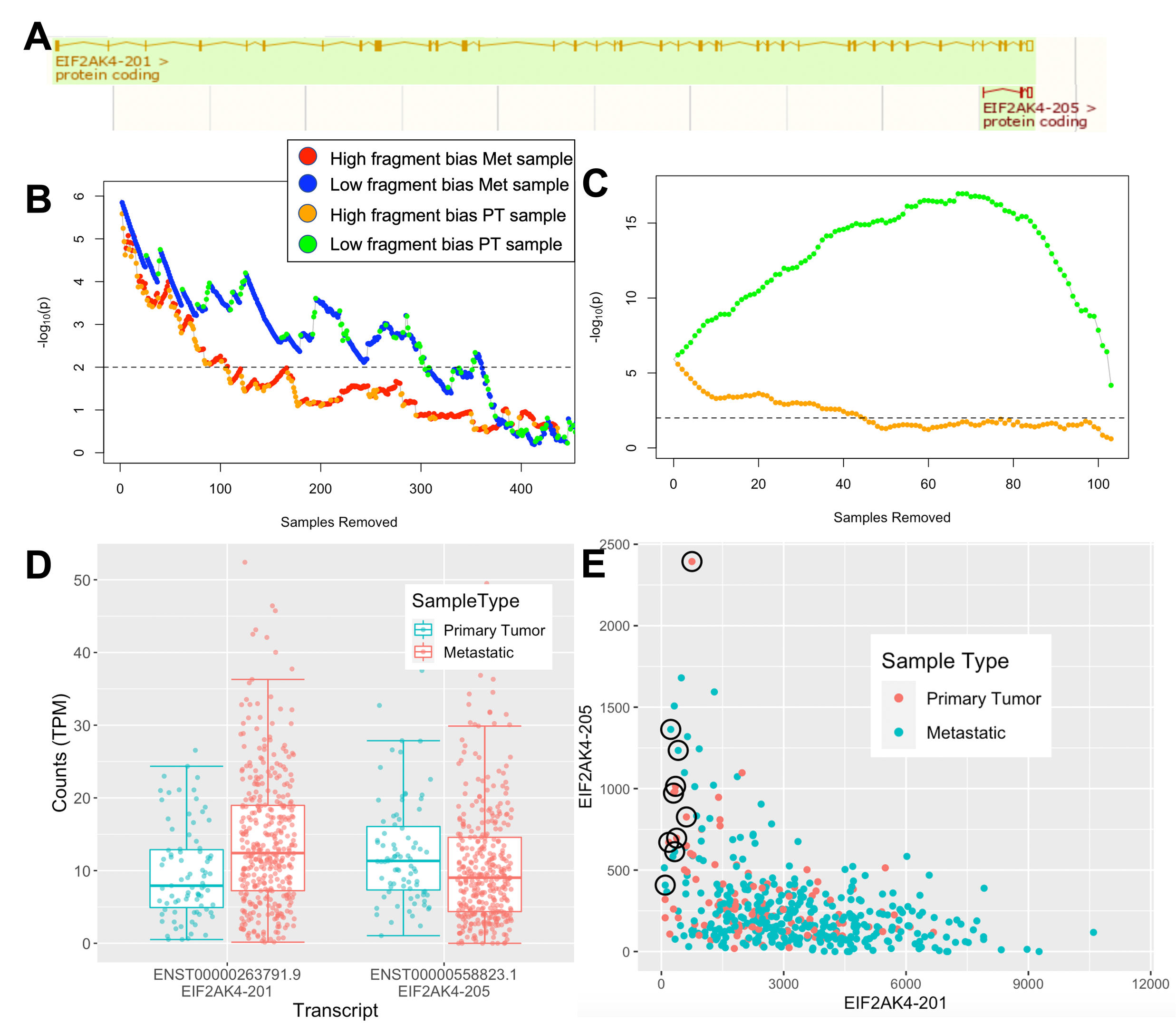

Supplement: S2 Fig — (A) Two protein coding isoforms of EIF2AK4, the full-length isoform (-201) and 3’ fragment (-205). (B) Change in DIR significance (-log10p) as samples are removed one-by-one in order of highest bias (red and orange dots) vs in order of lowest bias (green or blue dots). The significance drops faster when the high-bias samples are removed. The p-value here is calculated using the PCA method with the coin general independence test. (C) When only primary tumor samples are removed, the differences in p-values are even more disparate, indicating that high-bias primary tumor samples drive significance. (D) Box plots for the three isoforms with the highest number of normalized counts. Significance is driven by a higher amount of the full-length isoform in metastatic samples but a lower amount (on average) of the 3’ fragment. (E) Scatter plot of the raw counts of each isoform in each sample. Circled in black are the ten isoforms with the highest 3’ bias, indicated by high levels of the 3’ fragment and low levels of the full-length isoform. (TIF) [file pcbi.1010065.s002.tif]

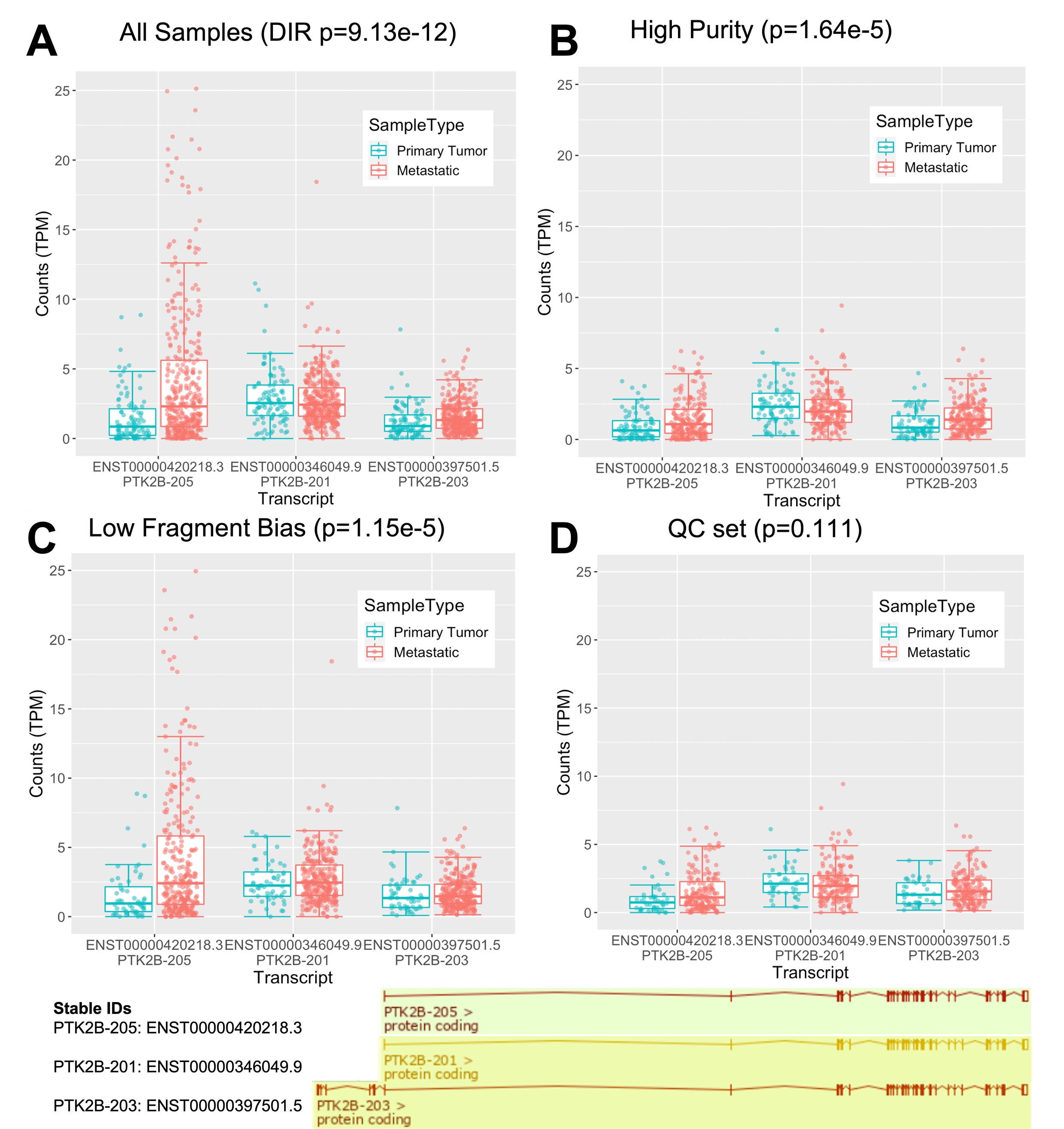

Supplement: S3 Fig — Expression of isoform PTK2B-205 in particular is driven by low-purity metastatic samples. Its expression drastically decreases when they are removed. Conversely, there is lower average expression of PTK2B-203 in primary tumor samples before samples with high 3’ bias are removed. This is likely due to the presence of more exons on the 5’ end, which will be undercounted in samples with 3’ bias. (TIF) [file pcbi.1010065.s003.tif]

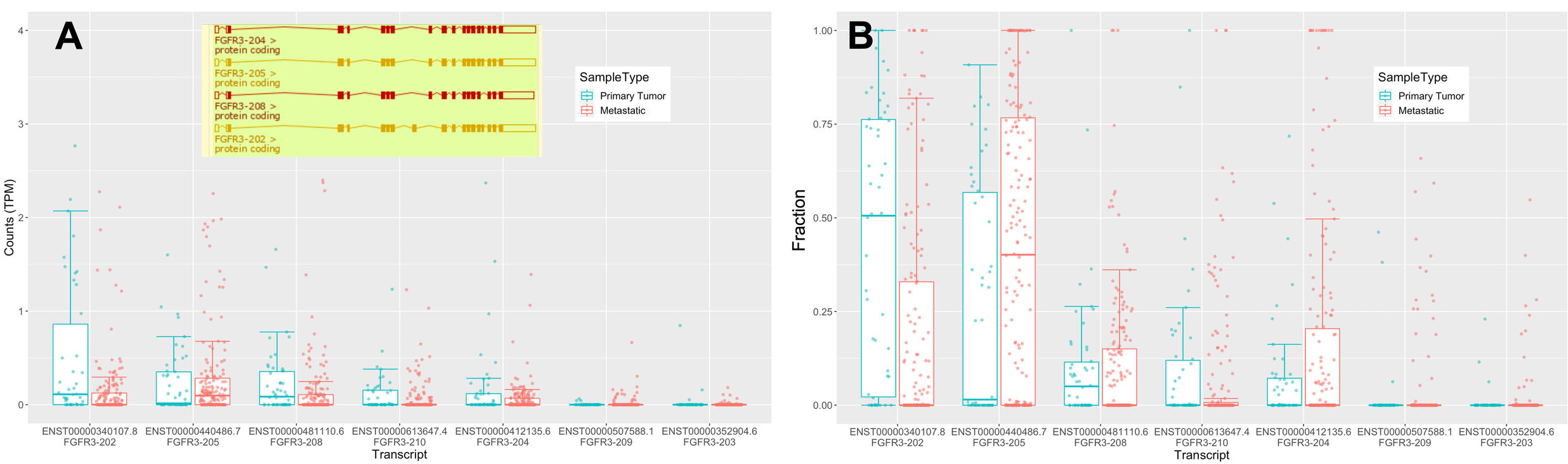

Supplement: S4 Fig — Plotted are (A) TPM counts and (B) fraction of all isoform counts for each sample. Although the trend is decreased expression, one isoform (FGFR3-205) has mildly increased expression, resulting in highly altered isoform ratios. (TIF) [file pcbi.1010065.s004.tif]

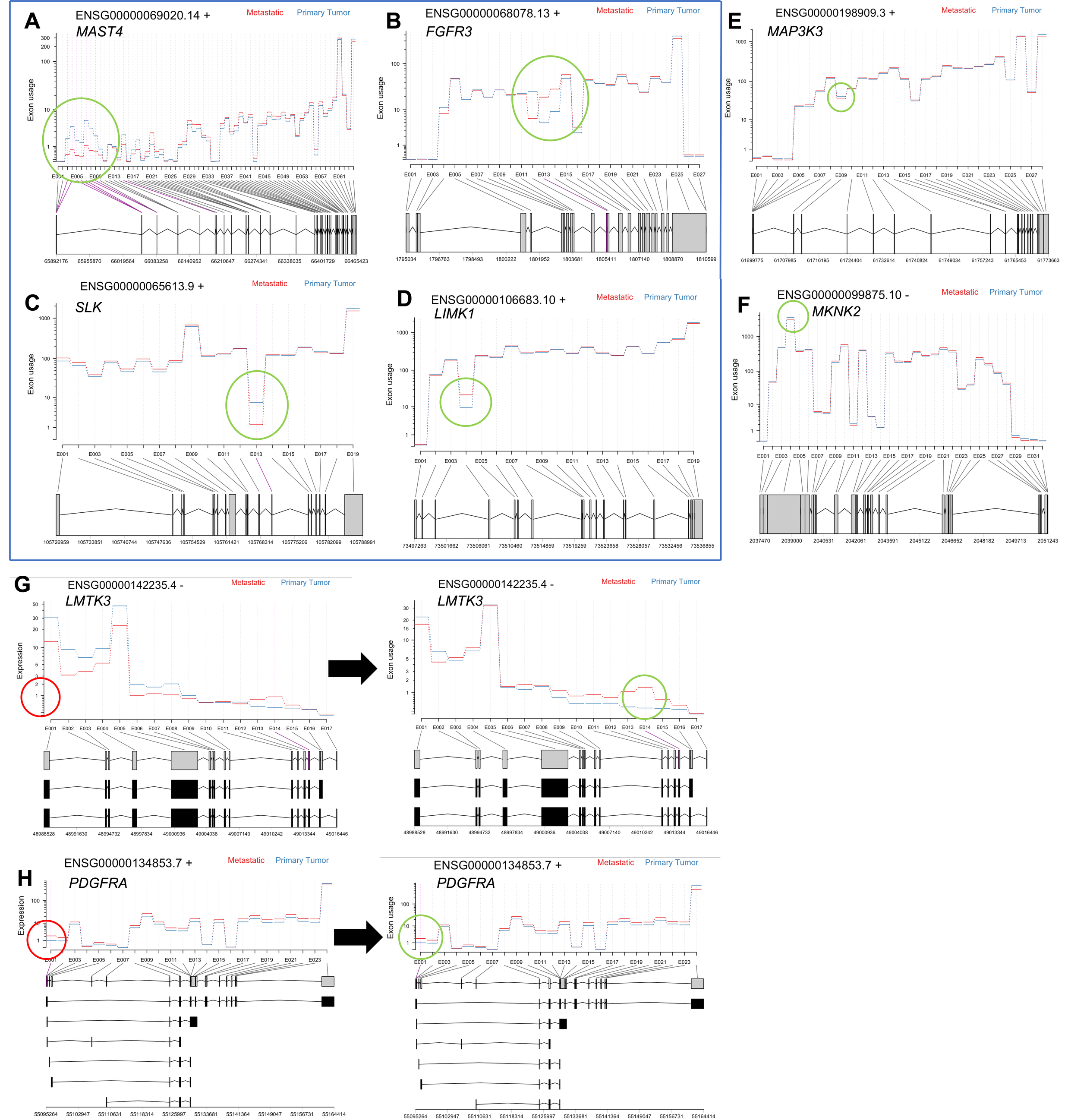

Supplement: S5 Fig — (A-D) DEXSeq confirmed DIR in three genes: MAST4, FGFR3, and SLK. The alternate promoter of LIMK1 was also significant before p-value adjustment. (E-F) The 3rd exon of MAP3K3 (bin 9) and MAPK-binding region of MKNK2 (bin 4) did not test significant with DEXSeq, even before p-value adjustment, despite testing as significant using exon junction alignment. (G-H) The 14th bin of LMTK3 and 1st bin of PDGFRA also tested as highly significant. However, these two exons have low expression (median ~1 count) so this result is likely due to noise and is unlikely to have biological relevance. (TIF) [file pcbi.1010065.s005.tif]

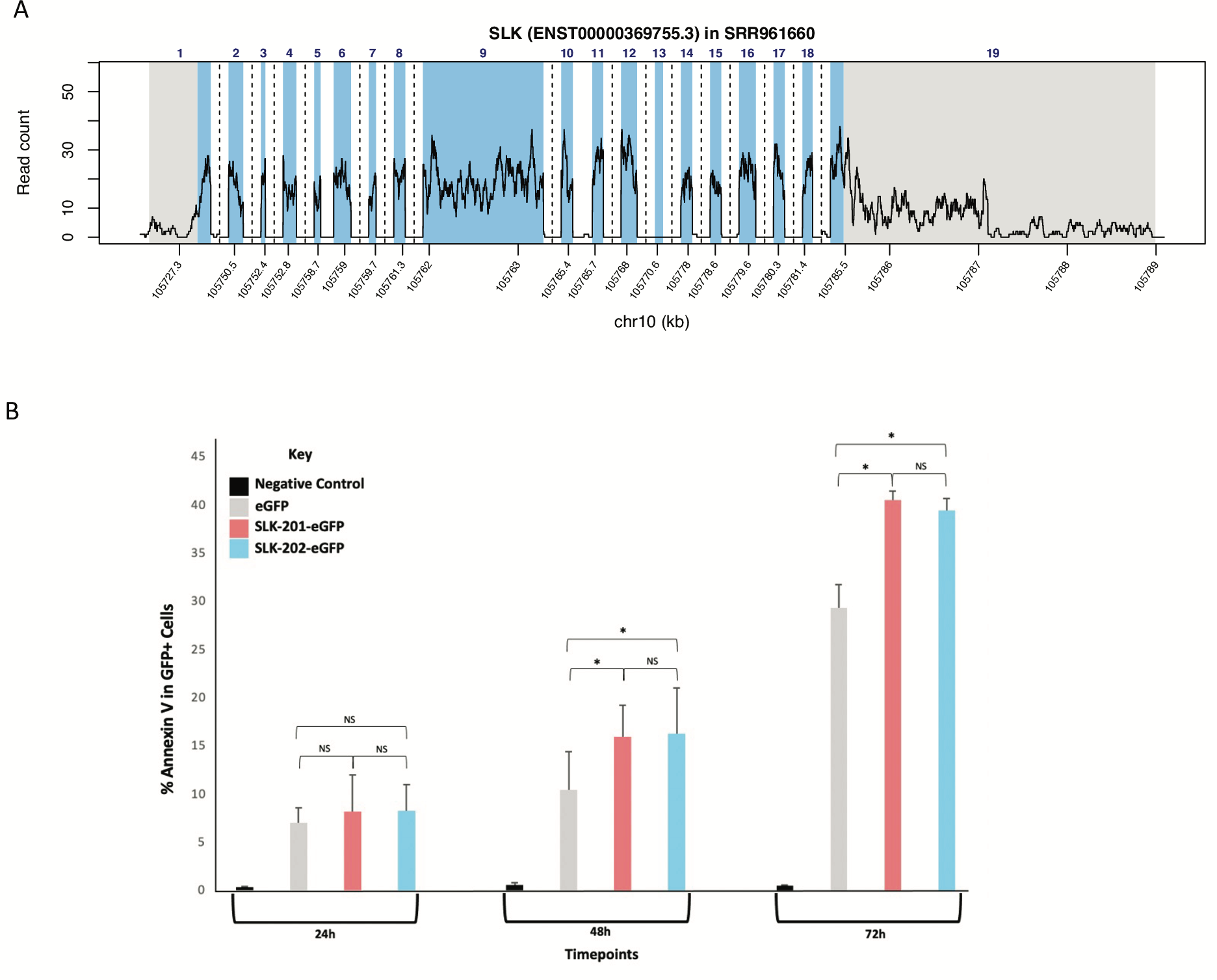

Supplement: S6 Fig — A) Plot of uniquely mapping sequence reads for A375 cells showing skipping of SLK exon 13. Original RNA-seq data are from the Sequence Read Archive SRR961660, https://www.refine.bio/samples/SRR961660. B) A bar graph showing annexin V staining over the 72h time course for 2 biological replicates. We see an increase in percent annexin V for both SLK isoforms at 48h and 72h compared to the eGFP-only control. All significant t-tests (*) had p-values < 0.05. All non-significant (NS) t-tests had p-values > 0.05. T-tests for the negative control were not included on the graph. (TIF) [file pcbi.1010065.s006.tif]

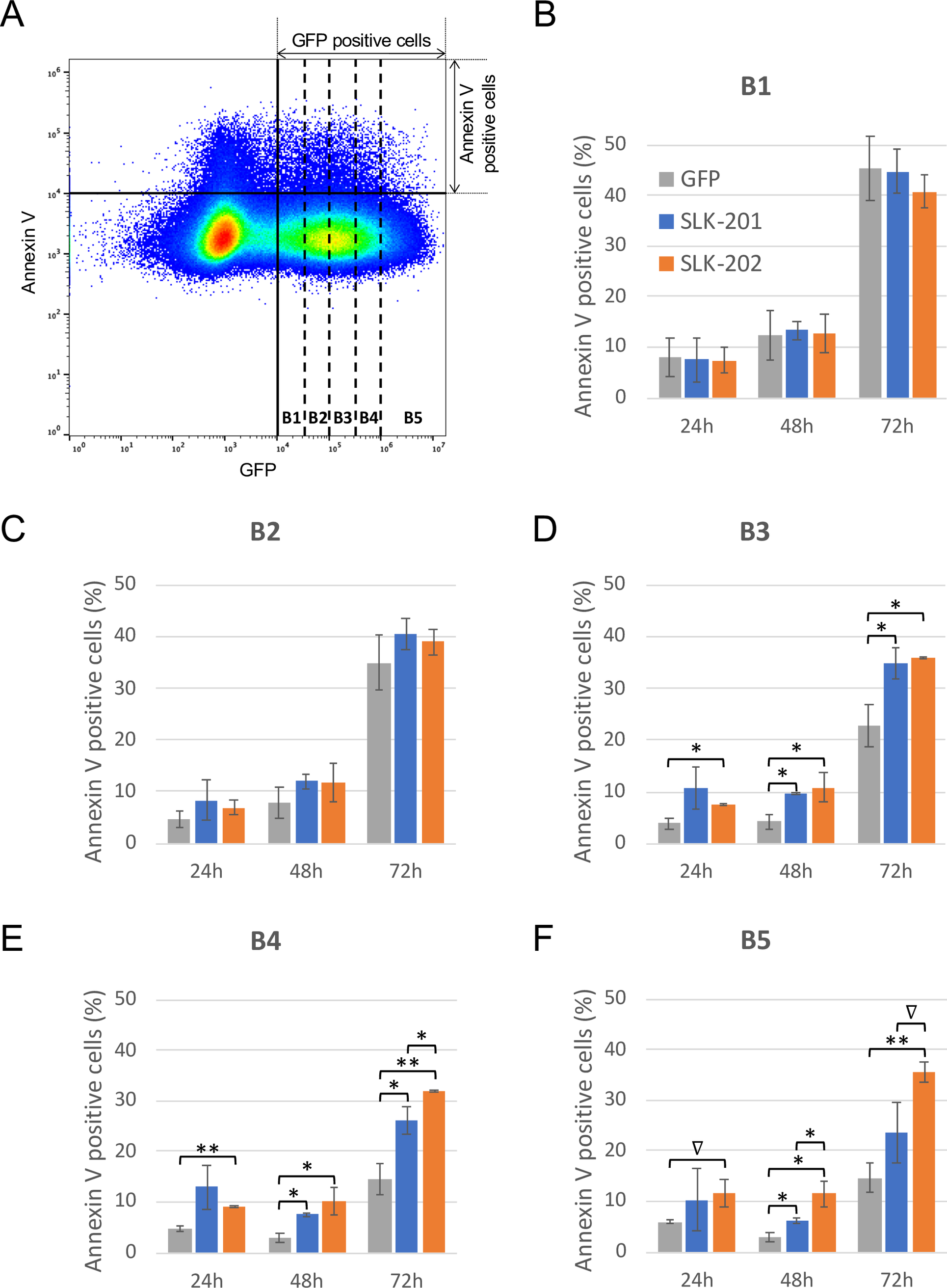

Supplement: S7 Fig — A) Illustration of thresholds used for determining annexin V positive cells (determined experimentally for each replicate, see Methods) in GFP-expressing cells (> 10^4 fluorescence units). Bins B1 through B5 represent cells with increasing GFP expression, and therefore also increasing levels of corresponding SLK isoform. Bin B5 is larger to accommodate the reduced number of cells expressing high levels of GFP. B-E) Comparison of incidence of annexin V positive cells in different constructs across different bins of increasing GFP expression (B1 –lowest, B5 –highest); **(p < 0.01), *(p < 0.05), ∇(p < 0.056). (TIF) [file pcbi.1010065.s007.tif]

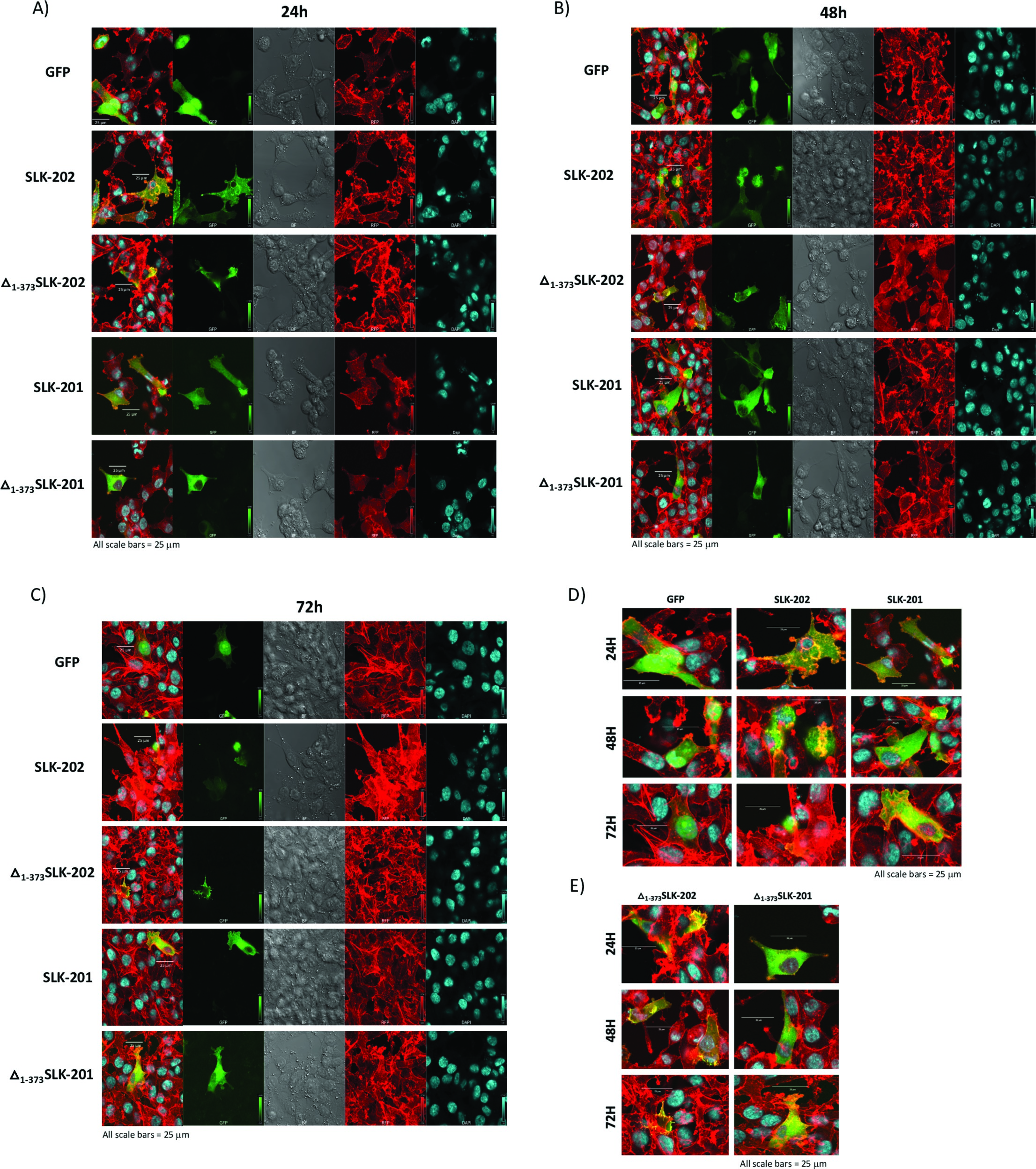

Supplement: S8 Fig — Representative images of each construct (green) at the A) 24-hour, B) 48-hour, and C) 72-hour timepoints. At each timepoint, cells were stained with DAPI (blue) and phalloidin (red). D) Merged channel images of eGFP-only, SLK-202-eGFP, and SLK-201-eGFP over the time course experiment. E) Merged channel images of △1-373SLK-202 and △1-373SLK-201 over the time course experiment. (TIF) [file pcbi.1010065.s008.tif]

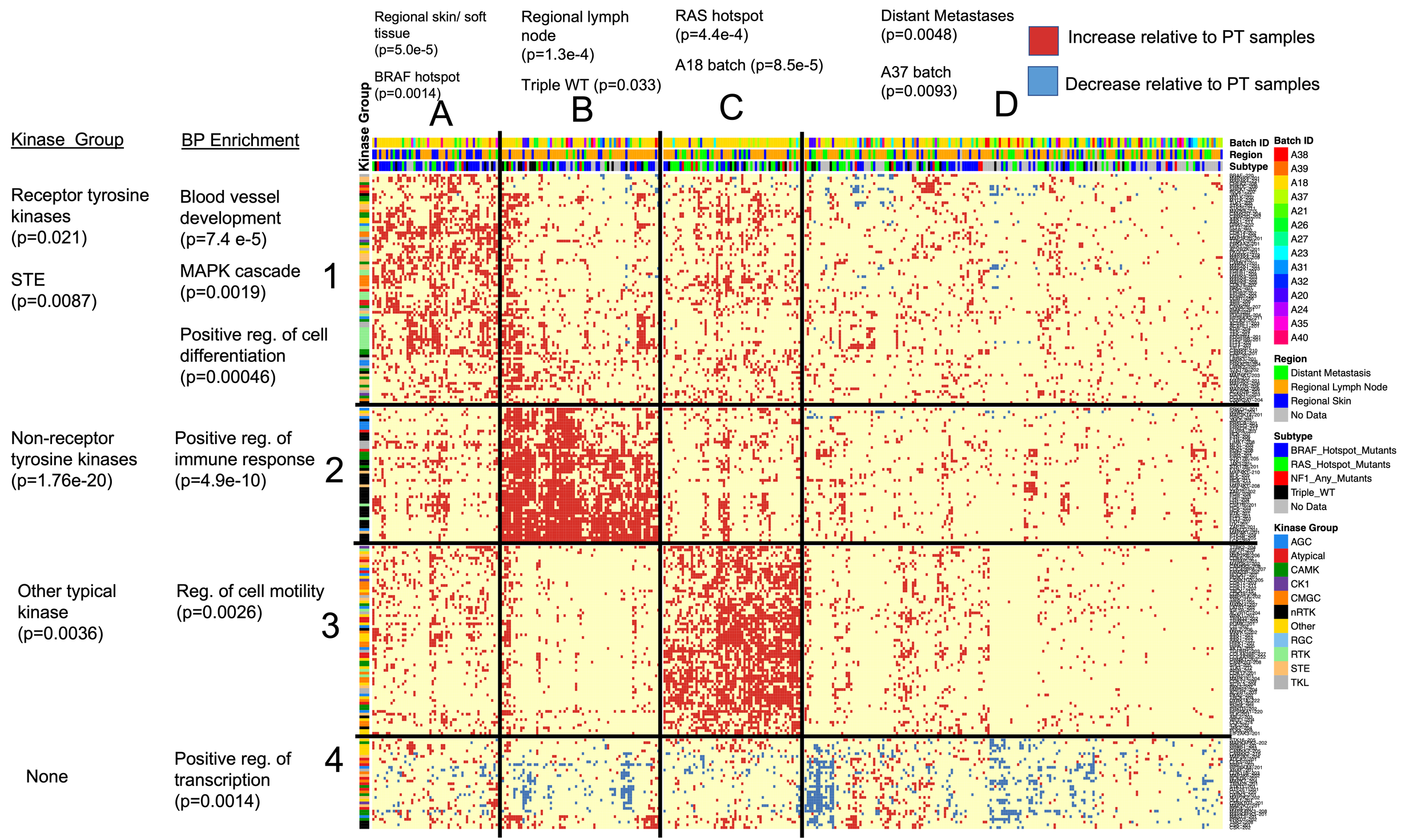

Supplement: S9 Fig — Red dots indicate increased expression in metastases (Quasi-Poisson GLM, p<0.05) while blue dots indicate decreased expression (p<0.2). Shown are the 367 metastatic samples (columns) and 235 isoforms that were altered in >13% of samples (rows). P-values were calculated using Fisher’s exact test. (TIF) [file pcbi.1010065.s009.tif]

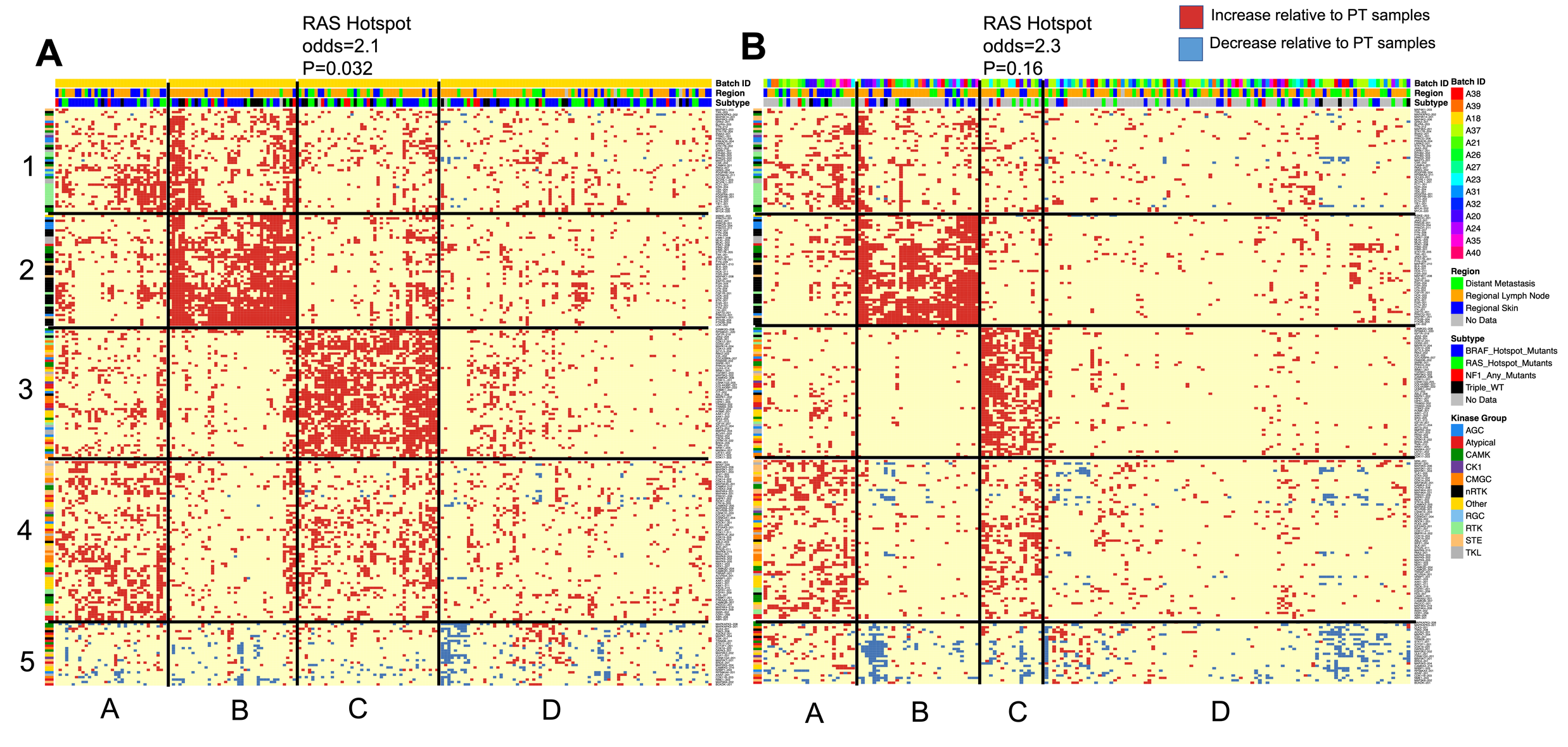

Supplement: S10 Fig — Isoform groups are the same as in Fig 8. Both sample subsets separated into four clusters comparable to Fig 8. (TIF) [file pcbi.1010065.s010.tif]

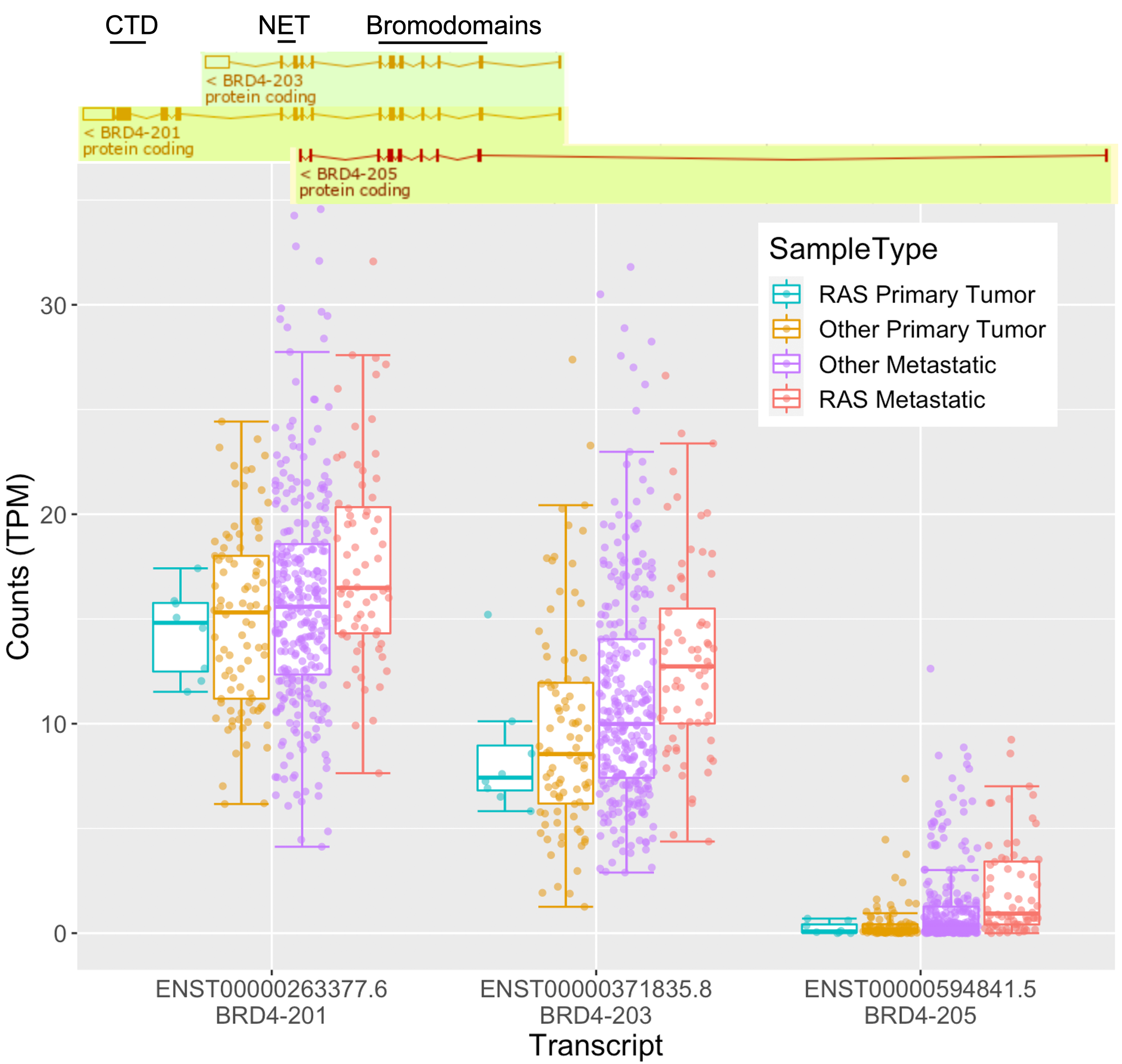

Supplement: S11 Fig — Although total BRD4 counts did not test as having significant DE between any group of primary and metastatic tumors, isoforms BRD4-203 and BRD4-205 have heightened expression in RAS-mutant metastatic samples. Exon junction analysis could not confirm these particular isoforms from sequence reads. (TIF) [file pcbi.1010065.s011.tif]

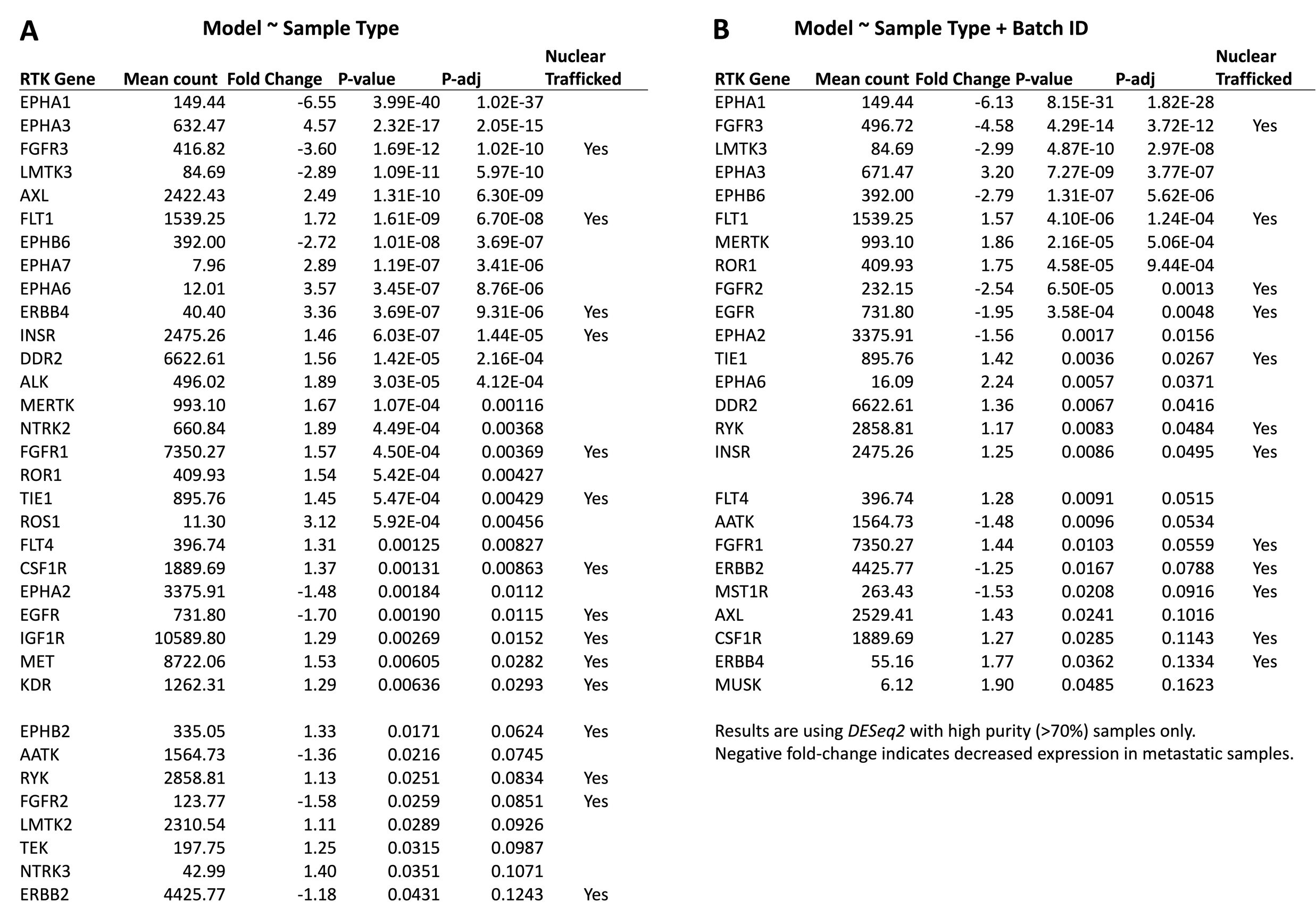

Supplement: S2 Table — (TIF) [file pcbi.1010065.s013.tif]
