# Supplementary material for: Characterization and clustering of kinase isoform expression in metastatic melanoma
Source: PLoS Comput Biol. 2022 May 13;18(5):e1010065. doi: 10.1371/journal.pcbi.1010065 (PMC9132324; doi:10.1371/journal.pcbi.1010065)
Supplement: Attachment — Submitted filename: ResponseToReviewers.VGedits.DH_V3.docx [file pcbi.1010065.s021.docx]

**Response to Reviewer Comments**

We sincerely thank each of the reviewers for their helpful and well-thought-out comments and suggestions. We have responded to each remark on a point-by-point basis.

Reviewer #1

1. My primary criticism is the choice to approach the isoform variation problem, when the data consists of short reads, with a full-length isoform estimation process. This approach also reveals a deficit of familiarity with the splicing literature—for analysis of short read data, differential isoform analysis is typically done by examining local splicing/promoter/alternative last exon variations within a gene, rather than trying to differentiate full-length transcript isoforms. Rather than trying to reinvent the wheel, the authors should perform such local splicing analysis using a number of software packages that were developed specifically for the purpose (e.g, DEXseq, rMATs, MAJIQ, MISO). In fact, DEXseq uses the same architecture that they have used here for total gene expression analysis, DESeq2.

We understand and appreciate this criticism. We are, in fact, aware of the current literature, for example, some of our analyses are similar to methods implemented in some newer tools, such as LeafCutter and LeafCutterMD, but are specifically tailored to our approach. Our results should be considered as obtained by intersecting the results of two different methodologies: one based on global isoform quantification, kallisto, and one based on the local analysis of split reads that are isoform specific. Combining these two methodologies offers several advantages, some of which might not be immediately obvious:

- Unlike some earlier tools (e.g. DEXSeq), kallisto is fast and highly scalable to many samples, such as the TCGA melanoma set that contains hundreds of samples. Its performance has been evaluated independently (Zhang *et al*. 2017. *BMC Genomics* 18: 583)
- The analysis of split reads we implemented here is a customized approach of the method used by LeafCutter that offers the advantages of examining reads highly specific to isoforms of interest, and of a localized analysis that accounts for various biases (e.g. 3’ bias). This is in line with the Reviewer’s observations (see below as well). Our customization also eliminates the influence of junctions that are not relevant for distinguishing two isoforms of interest that can happen in the standard LeafCutter analysis where normalization is done in clusters of junctions that share one of more exon ends. In our approach, we only consider two junctions that can distinguish between specific isoforms of interest.
- Access to estimated transcript counts allows further insights not possible through local analysis alone, such as the clustering analysis presented in Figure 8.
- Results confirmed by two different methodologies offer higher confidence, even if this comes at the expense of potential lower sensitivity. From the 14 cases reported in Table 4, we were able to confirm 6 cases (Figure 6 and 7) based on the analysis of split reads aligning at junctions specific to the isoforms of interest.
- The statistic we adapted here for analyzing kallisto estimates is a fast, easy to normalize approach that zeroes-in on exons known to be alternatively spliced, and produces a single p-value for each gene.

To accommodate the Reviewer’s criticism, we also performed a DEXSeq analysis, as one of the first and most popular methods for this type of analysis. We outline issues encountered by running the software, as well as the advantages of our approach. We discuss these analyses in blue text on Page 11 (Methods), Page 29 (Results), and Page 42 (Discussion). We add additional information in blue text in the Supplemental materials, on Page 7 (Full Results) and Page 8 (Figure S5).

DEXSeq is not optimal for our analysis for the following reasons:

- DEXSeq does not scale well to hundreds of samples and can be computationally very slow, especially during the estimate dispersions step.
- More importantly, DEXSeq poorly normalized exon counts when looking for splicing events. For example, the MAPK binding region of *MKNK2* (exonic bin 4) was reported to have decreased usage in metastatic samples, whereas bins 7-28 were shown as having increased usage to compensate (Figure S5). If bins 7-28 were superimposed for primary tumor and metastatic samples, bin 4 may have tested as significant. Similar events in the other genes led to low sensitivity.
- Some of DEXSeq’s most significant findings involved exons with very low counts that are likely false positives. For example, bin 14 of *LMTK3* tests as highly significant (p_adj_<0.05), but this exon had virtually no counts (median ~1 across samples). Nor it is an exon known to be alternatively spliced. Same with bin 1 of *PDGFRA*. (Figure S5)
- In the DEXSeq analysis, only 5 genes tested as significant after p-value adjustment (multiple test correction), including the two potential false positives mentioned above. Reasons for this low sensitivity include high dispersion in the data, poor normalization of exon counts, and having to adjust for p-values a large number of exonic bins (~14,000).
- Even before adjustment of p-values for multiple testing, DEXSeq failed to find some alternative splicing events we found with direct junction sequence alignment. These include the 3^rd^ exon of *MAP3K3*, MAPK binding sequence of *MKNK2*, and SH2 domain of *FES*.

2. Artifacts such as 3’ bias, which the authors correctly consider here in their analyses of full-length isoforms, are less of a problem in local splice variation, since the exons within a small segment of a transcript are subject to similar positional biases.

We agree with the Reviewer, and this is in fact one of the reasons for which we originally implemented local analysis of split reads, using a normalization procedure that accounts for various biases, including the 3’ bias. We note here, however, that we observed a stronger bias in primary tumor samples, which prompted us to eliminate the most affected samples, which was possible thanks to the size of the original dataset.

3. These approaches also allow for detection of novel splice variants in the tumor samples which as the authors correctly note may be unaccounted for in the annotation used here--the authors seem to be unaware of the literature post-2011 in stating “Currently, novel isoforms may be indirectly inferred by aligning reads to the nucleotide sequences of individual exons [72].”

We appreciate the Reviewer’s concern and have addressed it. As kallisto does not define novel isoforms, we intended to minimize this discussion. However, to avoid confusions and unwarranted assumptions, we have now removed this sentence from the manuscript, and on page 42 we point to tools that could be used for assessment of novel isoforms. "Specialized tools exist for this, such as *psiCLASS*, but this was not the focus of the present study.”

4. As stated in the discussion, “accuracy decreases as the number of gene isoforms increases”. This is because short reads from common regions of the isoforms of a single gene must be algorithmically distributed among the variants, which cannot be defined discretely in the absence of full-length transcript annotation.

Thus it is likely that many reads are allocated to non-existent isoforms and removed from the quantification of existing isoforms--further reason to switch to local analysis. It is very probable that a higher number of differentially expressed isoforms will be identified using one of the dedicated analysis packages, resulting in different outcomes—for example, the different gene profile in DE vs DIR could be a result of not detecting most of the alternative isoforms that are actually present, thereby underestimating the overlap.

Our confidence in kallisto estimates is based on an independent study that evaluated the accuracy of several tools (Zhang *et al*. 2017. *BMC Genomics* 18: 583, <https://bmcgenomics.biomedcentral.com/articles/10.1186/s12864-017-4002-1>). kallisto estimates (excluding very short transcripts) have a Pearson correlation of ~0.98 with “ground truth” numbers when genes have 1-5 isoforms, a correlation of ~0.97 for genes with 6-10 isoforms, and ~0.96 for genes with 11-15 isoforms. Our genes tested had a median of 5 isoforms and mean of 6.3 isoforms. Genes with significant DIR had a median of 5 and mean of 6.6 isoforms; the number of isoforms does not appear to affect whether the gene tests for DIR or not. We note here that none of the cases highlighted by us involve very short transcripts. All computational methods will deal with some level of inaccuracy, which is why we supplemented our method with direct junction sequence alignment as illustrated in Figure 6. The latter does not rely on isoform count estimation.

5. There is a section of the paper that is heavily oriented toward gene ontology, but even as the authors acknowledge the limitations of these analyses, there is much made of the gene groups identified. The manuscript would benefit from tailored gene ontology emphasizing the known functions of the kinase genes. Heavy reliance on gene ontology annotation (the groups are only as good as the annotations that produce them) may obscure other relevant biological processes. For example, what are all of the genes DE in “Ephrin receptor signaling pathway”, and are they all real examples of this pathway if you look at them individually?

The genes listed in the Ephrin receptor signaling pathway are part of that pathway. We added this sentence on page 18 "Significant Ephrin pathway genes included 7 ephrin receptors and downstream non-RTKs such as *ROCK1/2* (regulators of actin cytoskeleton, downstream of RhoA and *EPHA4[48]*), *PAK3* (downstream of *RAC1* and *EPHBs* and important for cytoskeletal reorganization in dendritic spines[49]) and *YES1* (oncogene downstream of *EPHA2* which induces cell proliferation and migration[50])."

We tested 1572 biological process annotations and looked into the specific functions of several results, including the Ephrin pathway (page 17, revised manuscript), *NME1* and *MAPKAPK3* (page 33), and the six genes where we found strong evidence of alternative splicing using junction sequence alignment (pages 37-38).

6. What is the relevance of nuclear trafficked kinases? This is never discussed.

On page 15 in Results we revised our text. “Of these 26 kinases, 11 are known to be internalized from the cell surface into the nucleus [44]: *FGFR1/3*, *FLT1*, *ERBB4*, *INSR*, *TIE1*, *CSF1R*, *EGFR*, *IGF1R*, *MET*, and *KDR*. Internalized receptors have been linked to cancer progression and resistance to therapy by, for example, activating DNA damage response pathways [45-47]”

7. The SLK overexpression experiment lacks controls. It is necessary to perform a western blot to determine expression levels of the two isoforms. Are the two isoforms distinguishable on blot? If so, is the endogenous protein ratio shifted in response to one or the other isoform overexpression? If not, looking at the RNA level could determine whether the endogenous splice variants are affected by overexpression. The endogenous pool could re-equilibrate the ratios between isoforms, obscuring the differences.

Further, the assay used here to determine apoptosis is not specific to apoptosis, it is a general cell viability assay. Better to use something like Annexin V staining.

More importantly, this experiment would be much more quantitative if done with a stable, inducible transgene (e.g. doxycycline) so as to remove the differences across the population due to variable transfection uptake and expression.

We have revised Figure S6 for clarity. We are using SLK-GFP fusions. Positive GFP signal indicates that the SLK constructs are being expressed. For the comparisons, we included no transfection control (indicated as “Negative control”) as well as an empty vector eGFP control (indicated as “eGFP”). In our revised experiments, included on Figure S6, we performed Annexin V staining as suggested and showed that the observed cell death is due to apoptosis. However, we could not distinguish a difference between the effects of the SLK isoforms with the transient transfection model. We agree that use of a stable, inducible transgene introduced into a background devoid of SLK activity would enable analysis without endogenous protein effects, but given the time would take such an experiment to complete, we believe it would be more appropriate for a future study. Currently, we limit ourselves to proposing such a study to verify the differences in function for the two isoforms.

8. There are a few instances of over-interpretation of data, e.g.:
   Pg 18, “this result suggests a distinct set of alterations are necessary for RAS hotspot mutants to become metastatic”; rather a distinct set of alterations is associated with RAS hotspot mutations in metastatic tumors. 
   A good portion of the discussion is highly speculative given the data presented here—for example, “The presence of distant metastases in Clusters C and D, which have little immune marker expression, suggest they may have evaded the initial immune attack in the lymph nodes” and “Perhaps cells that reach the lymph node have already evaded apoptosis.” These observations may suggest hypotheses to be further tested, but statements such as these represent multiple experimental steps beyond the data in question.

In response to the comments, we have removed or tempered these statements as seen by the blue text on pages 16 and 42 of revised main text. On page 16 we revised the text to state "this result suggests a distinct set of alterations is associated with metastases in RAS mutants.”

On page 42 we state "It is possible samples with this expression pattern, which includes many *RAS* mutants, may evade immune detection, which would explain this apparent mutual exclusivity. But it is also possible the low purity of these samples obscures increased expression of Group 3."

**Reviewer #2**

1. My main concern with the paper is that it is very difficult to follow which samples are being studied at each point, as the authors keep changing the dataset: from the complete dataset, to excluding samples due to low purity, over-representation of 3' ends or batch effects.
    
   While the reasoning behind each of these changes is scientifically sound and well-justified, it makes comparisons across experiments very difficult to follow. 
    
   For example, the authors make a good case for excluding some samples in the gene-expression analysis due to having low purity and this leading to biases caused by immune-infiltrate and not changes in the cancer cells. But then, they go back to study the entire dataset for the first differential isoform analysis, and then excluding some other samples due to other biases, making the comparison between the results of the isoform changes and differential expression very difficult to follow for the reader. 
    
   This could be solved, for example, by discussing the results of the DE and DIR experiments, with the same cohorts, side-by-side (i.e. the ones with the complete dataset, then the ones excluding the samples with low purity, then by hotspots etc.)

We thank the reviewer for the feedback about clarity of the organization. We intend to emphasize the DIR analyses and how they change among conditions, rather than to do a side-by comparison of DE and DIR. To clarify the organization, we now explain the structure of the paper at the start of the Results (page 13 of main text). This will hopefully make the Results easier to follow.

2. - Have the authors considered using other orthogonal data from TCGA to validate some of the findings? I am thinking particularly about the SLK hypothesis. I think it is great that the authors have tried to validate it in cell lines, and also that the negative outcome of the experiment should be reported, but maybe they could use gene expression or RPPA data to test whether the apoptosis pathway is more or less active depending on the SLK isoform being expressed.

We thank the Reviewer for this suggestion. We have now added the RPPA analysis, which had data available for 165 genes, including 33 kinases (page 19 main text, page 3 of supplement for figure S1). The following text is included in the revised manuscript on pages 19-20:

**Reverse Protein Phase Array (RPPA) Data**

We compared our results with an orthogonal dataset containing reverse protein phase array (RPPA) Data. Although isoform information was not available, 33 kinase genes had available RPPA data, wherein 14 genes (42%) had significant (p_adj_<0.05) differential expression (Figure S1, compared to 60 of 175 non-kinase genes (34%) (Table S5). The 14 genes include two RTKs, *ERBB3* and *KIT*. While the number of kinase genes covered by the RPPA data is too small for a signaling pathway enrichment analysis, a gene ontology analysis revealed that the 14 genes participate in vital biological processes related to cell growth and proliferation. In particular, the cell cycle regulatory genes including *EEF2K*, *PRKCD*, *PRS6KB1*, *CHEK2*, *MTOR* and *BRAF* were all upregulated in the metastatic group. These results corroborate that some of the kinase genes are also dysregulated at the protein level as tumors progress from primary to metastatic state. We did not find evidence that the significant kinases were related to apoptosis.

Because so few genes had RPPA data available, we chose to correlate *SLK* isoform expression with genes involved in apoptosis using normalized gene and isoform counts. Of our 538 kinases, 199 had a PANTHER annotation related to either apoptosis or “cell death”. *SLK* as a whole has strong correlations with *ROCK1*, *MAPK8*, and *TAOK1* (Spearman rho = 0.705, 0.668, and 0.609 respectively), all known to play a role in apoptosis. Testing the individual isoforms yielded different results: *SLK-201* (the short isoform overexpressed in metastatic samples) correlated with *CDK11A* (rho=0.704) and *AURKA* (rho=0.665), two more genes known to regulate apoptosis. *SLK-202*, the long isoform, had weaker correlations, but this is expected due to the fact that it has near-zero expression in many metastatic samples. Its strongest correlations were with *ABR* (rho=0.306) and *AURKB* (rho=0.258), two genes that had no correlation with *SLK-201.* A more in-depth examination of the apoptotic pathways activated by SLK is beyond the scope of this paper, but SLK expression did prove to have moderate-to-strong correlations with several apoptotic regulators. These results are now discussed in the Supplemental Results on page 9.

**Reviewer #3**

Minor comments 1. TCGA studies now incorporate RPPA (reverse phase protein arrays) analysis which will provide data on which proteins have increased expression. Could the author incorporate the protein expression analysis with the differential expression analysis to see how often differential expression correlates with increased protein expression. Furthermore these may provide a better snap shot of bona fide targets where increased expression correlates with detected increased protein expression for a subset of kinases. Are RTKs also over represented in RPPA analysis of metastatic samples?

In our response to **Reviewer #2, question 2,** we have added the RPPA analysis (see above and revised manuscript pages 19-20), which had data available for 33 kinases (page 19 main text, page 3 of supplement for figure S1). 14 of these kinases tested for significant DE using the RPPA data (Wilcoxon test). Only two RTKs were included in the 14 significant genes, including ERBB3 and KIT. It cannot be concluded that RTKs were overrepresented in the RPPA analysis based on the available data.

2. TCGA studies also incorporate survival data and is there a signature for metastatic tumors that may correlate with better or worse prognosis? Ie does increased expression of an RTK cluster portend poorer survival? Does decreased expression of RTKs predict better survival for example?

We appreciate the Reviewer’s suggestion and we now include survival curve analyses on Page 37 and Figure 9, with methods on Page 11. Immune infiltrate correlates with better survival, as does increased or decreased expression of some genes (though not to a great extent). These genes were not RTKs, although two of them are involved in electrolyte regulation.

Mutant-*BRAF* has non-significant increased expression in metastatic samples, as we now note in the main text (page 16). In response to this comment we investigated the correlation between mutant-*BRAF* and RTK gene counts, normalized by sample size factor. Overall, correlations were weak, with only one gene, *RYK*, having a Spearman correlation rho > 0.4. *AXL* and *MERTK* had no correlation (rho = 0.027 and 0.076 respectively in high purity *BRAF*-mutant samples). Correlation did not go hand-in-hand with differential expression with the exception of *FGFR3*, which had moderate negative correlation (rho = -0.25) and decreased expression in *BRAF* mutant samples (p-adj = 0.0012). Given that RTKs with high DE have low correlation with mutant-*BRAF* expression and vice versa, it appears that a change in mutant-*BRAF* expression does not drive DE of these genes.

3. It is very interesting the differential expression of RTKs is observed for the BRAF mutant subset of melanomas. In general V600 mutant melanomas often suppress signaling through other survival pathways to avoid cell death that can happen upon hyperactivation of numerous cellular proliferation and survival pathways. Inhibition of mutant BRAF relieves the suppression and often results in activation of upstream signaling cascades such as EGFR. Evaluation of Table S2 indicates there is a decrease in EGFR expression, which would be consistent with this observation. However, numerous RTKs have increased expression such as AXL. How does this correlate with mutant BRAF expression in BRAF mutant melanomas? Is mutant BRAF expression decreased? Having increased expression of known drivers such as MERTK in metastatic melanoma or RTKs that drive resistance to RAF inhibitors is very exciting and could drive therapeutic intervention strategies in the clinic – the authors should discuss the implications of these findings.

We now discuss these findings in page 2 the Supplemental Results.

4. For the SLK experiments, it would be informative to know if this is apoptosis caused by the two different SLK isoforms (sub-2n DNA content by FACS analysis for example). Furthermore it would be interesting if the SLK isoform lacking the coiled-coil domains actually promotes cell proliferation or migration for example, knocking out this isoform in a cell line expressing this shorter isoform would be informative.

We have repeated our experimental analysis to include measurement of apoptosis by Annexin V staining. The SLK constructs were causing significantly more apoptosis than the positive control. This result indicates that the apoptosis is coming from the SLK expression.

We thank the reviewer for the suggestion of isoform knockouts. As we responded to Reviewer 1, question #7, we would need to generate stable SLK clones lacking each isoform, and reintroduce one or the other to generate expression levels that more closely resemble those in actual tissue rather than having them overexpressed through transient transfection. The shorter isoform retains one coiled-coil domain, and thus can dimerize, but the kinetics may be slower, even though this might be compensated by the higher expression level associated with the transient transfection. This could explain why both isoforms induce apoptosis. As mentioned above, given the time would take such an experiment to complete, we believe it would be more appropriate for a future study. Currently, we limit ourselves to proposing such a study to verify the differences in function for the two isoforms.
