# Supplementary material for: Characterization and clustering of kinase isoform expression in metastatic melanoma
Source: PLoS Comput Biol. 2022 May 13;18(5):e1010065. doi: 10.1371/journal.pcbi.1010065 (PMC9132324; doi:10.1371/journal.pcbi.1010065)
Supplement: Attachment — Submitted filename: Holland_response03_02_22.docx [file pcbi.1010065.s022.docx]

Response to Reviewers

We thank the reviewers for their time and comments in helping to improve the manuscript content.

Reviewer #1: I am satisfied that the authors have properly addressed many of my criticisms concerning their differential splicing analyses. To the extent that differential isoform expression can potentially stratify tumors based on genomic subtypes, metastatic potential, and even prognostic indicators is a key finding.

The results of the sole experimental portion of the manuscript (SLK isoform effects on apoptosis) are disappointing, given that the underlying hypothesis of the study is that splicing isoforms can affect biological processes differentially. I wonder whether this experiment is being analyzed in the most appropriate way. In Fig S6 B, the FACS plots are shown for the eGFP control versus negative. The FACS plots for the two SLK-GFP fusions are not shown. If there is a difference in the function of the two SLK isoforms at different concentrations, it should be possible to compare the percentage of Annexin-positive cells within SLK-GFP expression bins (divided along the x-axis) between the two constructs. The prediction would be that SLK-202 would exhibit higher Annexin staining within lower GFP expression bins than SLK-201.

Without the isoform function data, the question is whether the differential isoform expression signatures constitute a significant advancement in classification/prognostication for melanomas.

We would like to thank the reviewers for the recommendation to further evaluate differences in *SLK* isoforms in our transfected cells. We now report differences in the percent of apoptotic cells and SLK localization based on isoform type. The reviewer's advice to bin the annexin V data by eGFP expression levels was insightful. We have updated Figure 7 to show the difference in percent of annexin V positive cells between *SLK*-202 (long) and *SLK*-201 (short) isoforms for bins of increasing eGFP fluorescence and different time points. We further report these data in Figure S7A.

In addition, we performed microscopy of metastatic melanoma cells transfected with *SLK*-202-eGFP (long) and *SLK*-201-eGFP (short). A new Figure 7E shows merged channel images of A375 cells transfected with eGFP-only, *SLK*-202-eGFP, and *SLK*-201-eGFP constructs at the 24-hour timepoint. Cells were stained with DAPI (blue) and phalloidin (red), along with GFP expression (green) and show the co-localization of *SLK*-202-eGFP with actin at the nuclear periphery, whereas *SLK*-201-eGFP has diffuse cytoplasmic staining at 24 hours post-transfection. We further illustrate staining patterns for 24, 48 and 72 hours in Figure S8.

We have added the following text to page 32:

We observed a significant increase in the percentage of annexin V positive cells at the 48h and 72h time points in both *SLK* constructs compared to the eGFP-only control, supporting the hypothesis of *SLK*-induced apoptosis (Figure S6B). We observed no significant difference between the effect of the two *SLK* constructs when comparing all GFP positive, annexin V positive cells (Figure S6B). However, when eGFP expressing cells were divided into bins, B1 – B5, corresponding to increasing expression levels of eGFP-fusion constructs (Figure S7A), we observed an increase in the difference of percent annexin V positive cells between *SLK*-202 and *SLK*-201 at the 48h and 72h time points (Figure 7D, S7A). These data indicate that the long *SLK* isoform (*SLK*-202) induces apoptosis at a higher rate. This finding corresponded to increasing construct expression levels, indicating that the functional impact of the longer isoform could be detected only at higher expression levels and longer timepoints (Figure S7B-F).

We also found that *SLK*-202 co-localizes with actin filaments more strongly than *SLK*-201 or the eGFP-only control (Figures 7E, S8A-D). At 48h, the *SLK*-202 transfected cells begin to lose their structure, and by 72h, the cells have mostly detached. Since the N-terminus of *SLK* contributes mainly to the cell death [57], we removed the N-terminal 373aa of *SLK*-202 (Δ_1-373_*SLK*-202). Δ_1-373_*SLK*-202 had a unique localization to actin filaments along the periphery of the cell (Figure S8A-C, E). Δ_1-373_*SLK*-201 looked similar to *SLK*-201. These results suggest that *SLK*-202 localizes to and potentially disassembles actin more effectively in metastatic melanoma compared to *SLK*-201, consistent with *SLK*-202 being more apoptotic in our binned data (Figure 7D).
